# Understanding the ancient classic and famous prescriptions *via* the property of Chinese materia medica

**DOI:** 10.3389/fphar.2025.1551531

**Published:** 2025-05-12

**Authors:** Dan Qin, He Zhang, Bin Du, Hui Wang, Ligang Liu, Yun Wang

**Affiliations:** ^1^ School of Chinese Materia Medica, Beijing University of Chinese Medicine, Beijing, China; ^2^ Department of Pharmacy, Medical Supplies Center of Chinese PLA General Hospital, Beijing, China; ^3^ Beijing International Center for Mathematical Research, Peking University, Beijing, China; ^4^ School of Mathematical Sciences, Peking University, Beijing, China; ^5^ Institute of Therapeutic Innovations and Outcomes (ITIO), College of Pharmacy, The Ohio State University, Columbus, OH, United States

**Keywords:** ancient classic and famous prescription, Chinese materia medica, property of Chinese materia medica, medication pattern, feature extraction, combinatorial optimization

## Abstract

**Background:**

Ancient classic and famous prescriptions (ACFPs), derived from traditional Chinese medicine (TCM) classics, are widely utilized due to their precise therapeutic effects and distinctive clinical advantages. Existing research predominantly focuses on individual prescriptions, and there is lack of systematic exploration of medication patterns within the official ACFPs catalog. The property of Chinese materia medica (PCMM), a multidimensional representation of medicinal properties, offers a novel perspective for systematically analyzing TCM formulas.

**Objective:**

In this study, we aim to investigate the implicit medication patterns of ACFPs from the PCMM perspective, establish a feature extraction model based on the property combination of Chinese materia medica (PCCMM), and evaluate its effectiveness in representing and reconstructing ACFPs.

**Methods:**

Based on the Chinese Pharmacopoeia (ChP), we constructed a CMM–PCCMM network as the forward feature extraction process. We formulated the backward process as a constrained combinatorial optimization problem to rebuild ACFPs from their PCCMMs. We evaluated the performance of PCCMM in reconstructing ACFPs using the Jaccard similarity coefficient. Furthermore, we tested the capability of PCCMM to distinguish ACFPs from random pseudo-formulas and classify ACFPs according to deficiency syndromes. Finally, we conducted frequency analysis, association rule analysis, distance analysis, and correlation analysis to explore the implicit medication patterns of ACFPs based on PCCMM.

**Results:**

Numerical experiments showed that PCCMM effectively represented and reconstructed ACFPs, achieving an average Jaccard similarity coefficient above 0.8. PCCMM outperformed the nomenclature of CMM in distinguishing ACFPs from random pseudo-formulas and classifying deficiency syndromes. Frequency analysis revealed that high-frequency CMMs were mainly tonic medicines, whereas high-frequency PCCMMs predominantly mapped to the even–sweet–spleen meridian. The association rule analysis based on PCCMM yielded significantly more implicit compatibility rules than CMM alone. Distance and correlation analyses identified synergistic CMM pairs and PCCMM pairs, such as *Jujubae Fructus* (Dazao) and *Zingiberis Rhizoma Recens* (Shengjiang), which is consistent with clinical experience.

**Conclusion:**

The PCCMM-based feature extraction model provides a quasi-equivalent representation of TCM formulas, effectively capturing implicit medication patterns within ACFPs. PCCMM outperforms traditional CMM methods in formula reconstruction, classification, and medication pattern mining. This study offers novel insights and methodologies for systematically understanding TCM formulas, guiding clinical application, and facilitating the design and optimization of new TCM formulas.

## 1 Introduction

The ancient classic and famous prescriptions (ACFPs), derived from the ancient traditional Chinese medicine (TCM) books, are widely utilized TCM formulas known for their precise curative effects, distinctive features, and notable advantages (Qian et al., 2019). The research, development, and utilization of ACFPs are vital sources for R&D of new drugs in TCM (Xue-Mei et al., 2019). As one of the breakthroughs in the inheritance and development of TCM, the research of ACFPs has been a hotspot in recent years (Su et al., 2024) after the National Administration of Traditional Chinese Medicine and the National Medical Products Administration published the catalog of ACFPs in 2018 (first batch) and 2023 (second batch). Some scholars systematically explored the historical evolution (Bing et al., 2019) of the formulas from the source, composition, dosage, processing, clinical application, function interpretation, and decocting method by comprehensive collation of ancient and modern literature on ACFPs (Li S. et al., 2022). Some scholars researched chemical profiling and quantification of the ACFPs to provide a solid basis for quality control and mechanisms (Zhou et al., 2023). Some studies examined the active components (Wang S. et al., 2021), efficacy (Xiao et al., 2022), and pharmaceutical mechanism (Xia et al., 2023) underlying the effects of ACFPs on some particular diseases using network pharmacology analysis and molecular docking in combination with experimental validation. On this foundation, some studies have found the unexplored therapeutic effects and mechanisms of a particular ACFP (Hao et al., 2022). However, most current studies on ACFPs focus on a specific prescription, and there is still a gap in the systematic regularity study of the catalog of ACFPs issued by the Chinese government.

It is conducive to providing references for evaluating TCM formulas or designing new formulas through the systematic study of the formation and medication patterns of the ACFPs to explore the scientific connotations of the formulas implied by their broad application, safety, and efficacy. To find the medication patterns in the formulas, some studies tried to explore the scientific connotations of the TCM formulas by data mining, such as frequency analysis (Yang et al., 2021), cluster analysis (Guo et al., 2022), and association rule analysis (Liu et al., 2023). Wu and Guo (2025) offered the potential for uncovering commonalities through the analysis of 2,344 prescriptions for pox treatment, and Xue et al. (2024) have employed data mining methods to analyze the medication patterns in the treatment of vascular dementia. However, these studies often focus on the medication patterns of a specific disease. Machine learning and deep learning techniques, such as support vector machines (SVM) (Jin et al., 2020) and graph convolutional networks (GCN) (Zhao et al., 2022), have been widely applied in the design of new TCM formulas, without being restricted to specific diseases. However, these studies often treat medication patterns as black boxes and rely on large-scale datasets (Li D. et al., 2022). Due to the wide variety of names and types of Chinese materia medica (CMMs), we often get a poor reproduction rate of elements in data mining in a limited-scale and nonspecific disease dataset. The sparsity of the signals makes it difficult to dig out the medication patterns of TCM formulas or generate a new TCM formula based on a small sample dataset of known TCM formulas. Feature extraction can transform raw data into meaningful information, facilitating enhanced data reuse through a standardized process (Lamer et al., 2022). For example, Miao et al. (2023) employed an improved ConvNeXt network to extract features for constructing a TCM identification model. Gong et al. (2023) integrated feature extraction with a multi-label deep forest model, which achieves efficient processing of syndrome differentiation in TCM. Therefore, a more efficient representation of TCM formulas than CMMs is needed to enhance the mining of medication patterns, subject to the low repetition rate of CMMs in small TCM formula datasets.

According to the property theory of CMM (PTCMM), the property of CMM (PCMM) is a multi-dimensional systematic representation of the basic properties and characteristics of the CMM’s efficacy (Qiao et al., 2022). Considering the standardization and authority of the Chinese Pharmacopoeia (ChP), the research studies about database construction and data mining related to the PCMM are predominantly based on the data extracted from the ChP, such as ETCM (Zhang et al., 2023a) and FordNet (Zhou et al., 2021). The core components of the PCMM include five herb properties (cold, warm, even, cool, and hot), seven herb flavors (bitter, pungent, sweet, sour, astringent, salty, and bland), and 12 herb meridian tropism (liver meridian, lung meridian, spleen meridian, stomach meridian, kidney meridian, heart meridian, large intestine meridian, small intestine meridian, Sanjiao meridian, bladder meridian, pericardium meridian, and gallbladder meridian) (Zhang et al., 2023b). Some research workers have considered the PCMM as a multidimensional systematic feature of the CMM (Qiao et al., 2022; Zhang et al., 2023b), but the feature extraction process from ACFPs to PCMM remains unclear. Based on these core components of PCMM, our lab has proposed a systematic view of PCMMs called the property combinations of CMM (PCCMM) (Hu et al., 2016) by coupling the elements from these three sets, which provides new ideas for the representation of CMMs in the TCM formulas.

**FIGURE 1:**
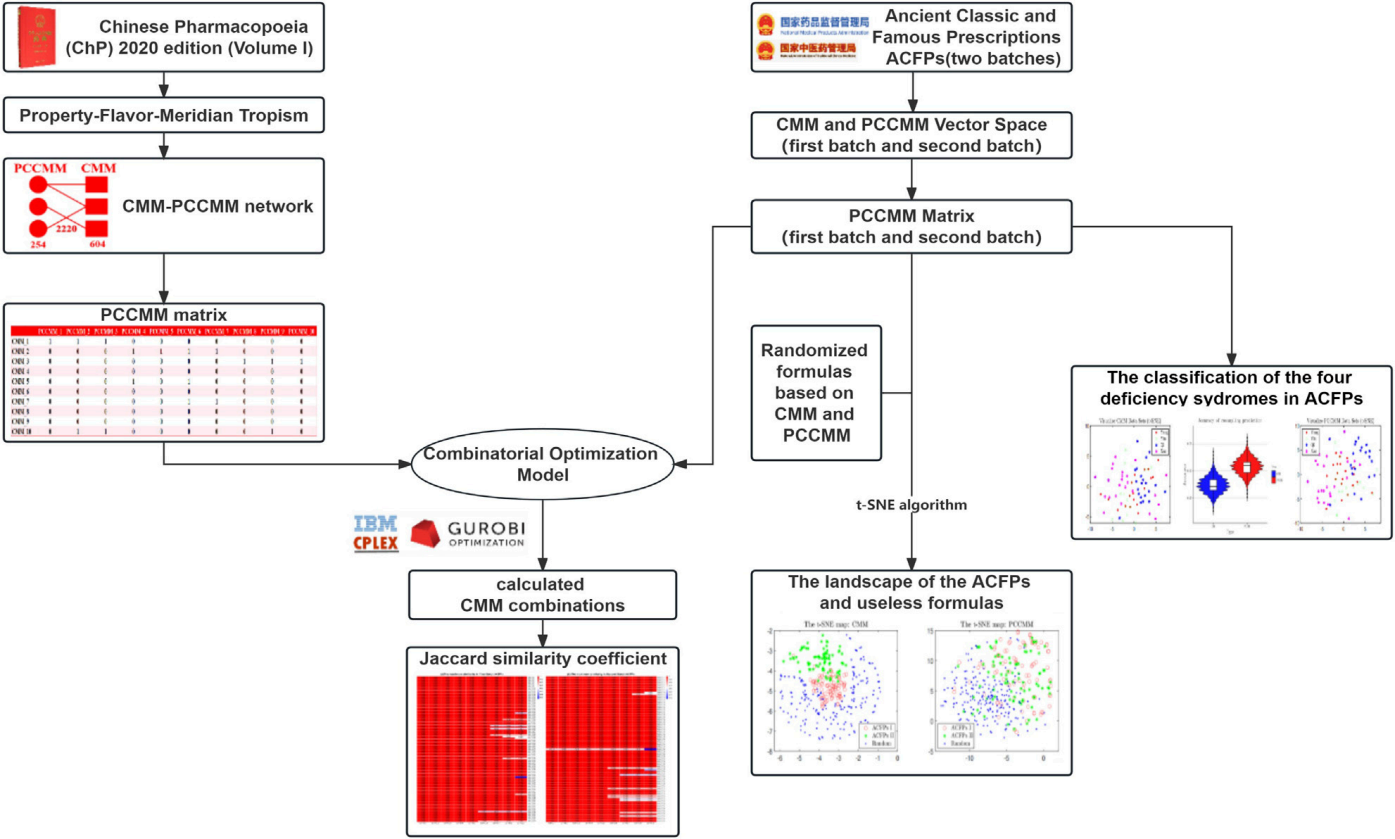
Flow diagram illustrating the process: the left side depicts the preprocessing of CMM and PCCMM data and the construction of the PCCMM matrix, which defines the combinatorial formula model in the center. The right side shows a series of numerical experiments conducted on the ACFP dataset.

In this study, we have introduced a novel, relatively low-dimensional representation of TCM formulas by feature extraction and employed this approach to analyze the implicit medication patterns of ACFPs. Figure 1 shows the flow diagram of the work. This study commences with the construction of a CMM–PCCMM network and PCCMM matrix as the forward feature extraction process. As for the backward process, utilizing the framework of compressive sensing, we introduce the combinatorial optimization problem and develop a combinatorial formula model based on PCCMM. With the ACFPs serving as a test set, we proceed to rebuild the ACFPs from the PCCMM. The performance of PCCMM in measuring the composition of TCM formulas is then evaluated based on its reconstruction capabilities. To further investigate the quasi-linear measurement capabilities of PCCMM for TCM formulas, we also test its proficiency in separating ACFPs from random pseudo-formulas and its ability to distinguish the deficiency syndromes associated with the ACFPs. Building upon this foundation, we conducted frequency, association rule, distance, and correlation analyses on ACFPs using PCCMM to uncover the underlying medication patterns.

## 2 Materials and methods

### 2.1 Data sources and preprocessing

#### 2.1.1 The CMM and PCCMM information

The CMM and PCMM information in this study was derived from the current ChP 2020 edition (volume I) (Chinese Pharmacopoeia Commission, 2020). As introduced in Section 1, a PCCMM is a triplet following the “property–flavor–meridian tropism” rule, where the three coupled labels are selected as representatives of PCMMs. For simplicity, throughout the rest of the paper, we will use the term “PCMM” to refer exclusively to these three labels: property, flavor, and meridian tropism. To begin with, we define the PCCMM set of a CMM as the collection of PCCMMs that includes all possible “property–flavor–meridian tropism” triplets subject to the PCMM of the CMM. For example, the property of *Ephedra sinica* Stapf (Mahuang) (Zheng et al., 2023) is warm, the flavor is bitter and pungent, and the meridian tropism is the lung meridian and bladder meridian based on ChP. Thus, there are four corresponding PCCMMs, namely, warm–bitter–lung meridian, warm–bitter–bladder meridian, warm–pungent–lung meridian, and warm–pungent–bladder meridian, which together form the PCCMM set of the CMM, *Ephedra sinica* Stapf. In general, if the PCMM of a CMM includes $n_{1}$ properties, $n_{2}$ flavors, and $n_{3}$ meridian tropisms, the resulting PCCMM set consists of a total of $n_{1}*n_{2}*n_{3}$ triplets. We denote the space of CMMs and PCCMMs as $I=\left\{a_{1},a_{2},\ldots ,a_{N}\right\}$ and $J=\left\{b_{1},b_{2},\ldots ,b_{m}\right\}$ , respectively. Here, each $a_{i}$ corresponds to a CMM listed in ChP with complete “property–flavor–meridian tropism” PCMM information. We found a total of 604 CMMs that satisfy the condition, that is, N=604 (Supplementary Table S1).

In practice, based on the dataset involved, one can specify the scope of CMM and the size of N for efficient computation. We will return to this issue in Section 2.2.4. Each $b_{i}$ is a triplet that resembles a possible PCCMM. Theoretically, as there are five herb properties, seven herb flavors, and 12 herb meridian tropisms, we can form 5×7×12=420 different triplets. However, only 254 PCCMMs are observed from the CMM dataset based on ChP, and we fixed m=254 throughout the paper (Supplementary Table S2).

We also conducted observational tests using data from the 2015 edition of the ChP. Compared to the 2020 edition, the 2015 edition includes three additional CMMs with complete “property–flavor–meridian tropism,” that is, PCMM, information: *Aristolochiae Fructus* (Madouling), *Manis Squama* (Chuanshanjia), and *Aristolochiae Herba* (Tianxianteng). As a result, the total number of CMMs with complete PCMM records in the 2015 edition amounts to 607 (see Supplementary Table S10), covering a total of 254 PCCMMs, which is the same as the 2020 edition. Upon a detailed comparison, we observed that the fields “property–flavor–meridian tropism” for the shared 604 CMMs recorded in the 2015 and 2020 editions are identical. This consistency ensures that the core PCMM information remains stable across the two editions, providing a reliable foundation for further computational and experimental studies.

#### 2.1.2 Incompatible CMM pairs

Revealing the connotation of the compatibility of CMM is a requirement for the modernization of TCM (Gao et al., 2023), so we need to avoid contraindications of the CMMs in the candidate formula; for example, the “eighteen incompatible medicaments” theory in TCM (Chen et al., 2019). In particular, according to the “Zhong Yao Pei Wu Jin Ji” (Duan, 2019), we extracted 89 incompatible CMM pairs (Supplementary Table S3), which will not appear simultaneously in the subsequent model calculations.

#### 2.1.3 The ACFP dataset

We extracted 93 and 85 prescriptions from the two batches of ACFP catalogs (Supplementary Tables S4, S5), respectively, published by the National Administration of Traditional Chinese Medicine and the National Medical Products Administration such that the CMMs involved are within the CMM space. Table 1 provides detailed information about the selected prescriptions.

**TABLE 1:** Number of prescriptions, CMMs, and PCCMMs of the two ACFP datasets. Repeated occurrences of CMMs or PCCMMs are not counted in the “unique” columns.

	Total	Selected	CMMs involved		PCCMMs involved	
Catalog	Prescriptions	Prescriptions	Unique	Sum	Unique	Sum
First batch	103	93	155	655	156	2,985
Second batch	93	85	136	511	158	2,243
Summary	196	178	196	1,166	176	4,026

Notice that a TCM formula, qualitatively, is a collection of CMMs. Thus, one can extend the concept of PCCMM sets to TCM formulas and even general CMM combinations by combining all the PCCMM sets of the involved CMMs. Thus, we obtain a map from ACFPs to their PCCMM sets.

### 2.2 Feature extraction of the ACFPs based on PCCMM

#### 2.2.1 The framework of compressive sensing

In this section, we review some fundamental concepts of compressive sensing to mathematically interpret the PCCMM matrix and combinatorial formula model introduced in Section 2.2.2 and Section 2.2.3. Although the compressive sensing framework offers mathematical foundations for the CMM–PCCMM network and the combinatorial formula model to be introduced in Section 2.2.2 and Section 2.2.3, these models are primarily driven by the feature extraction process from ACFPs to PCMM, with all variables holding clear pharmacological significance.

The development of compressive sensing theory started from the initial work by Emmanuel J. Candès, Justin Romberg, and Terence Tao (Candes et al., 2006) along with David Donoho’s study (Donoho, 2006). The success of compressive sensing depends on the sparsity or compressibility of the signal, either in its natural state or over a known basis. As a result, one can recover the essential information within few measurements of the observation by solving an underdetermined linear system of equations. For the mathematical theory and applications of compressive sensing, refer to the studies by Foucart and Rauhut (2013) and Rani et al. (2018), respectively.

In the compressive problem, the observed data $y\in R^{m}$ are connected to the signal of interest $x\in R^{N}$ *via*

$$y=Ax,\;A\in R^{m\times N},$$ (1)

where the matrix A models the linear measurement process. We assume m<N ; that is, the linear system in Equation 1 is underdetermined, which means there is no unique solution of x given a measurement y . In other words, it is impossible to recover x from y without additional information. The key saver is the sparsity assumption of x . In particular, the vector x is s -sparse if at most s of its entries are nonzero, that is,

$$\|x{\|}_{0}≔card\left(supp\left(x\right)\right)\leq s,$$

where card denotes the cardinality and supp(x) , called the support of vector x , is the index set of all nonzero entries of x . The sparse recovery problem corresponds to the following $\ell_{0}$ -optimization problem:

$$\min\|z{\|}_{0},\;\text{subject to }Az=y.$$ (2)

As solving Equation (2) is NP-hard in general, in practice, we consider the convex relaxation of Equation 2 given by

$$\min\|z{\|}_{1},\;\text{subject to }Az=y,$$ (3)

where $\|\cdot {\|}_{1}$ is the sum of the entries’ absolute value. In Equation 3, we treat the linear process in Equation 1 as hard constraints; this differs from the regression problems such as LASSO. The conditions on the matrix A that ensure the exact reconstruction of the original sparse vector x from the solution of Equation 3 are known as the null space property condition (Foucart and Rauhut, 2013).

#### 2.2.2 Building the CMM–PCCMM network and the PCCMM matrix

In this section, based on the compressive sensing framework reviewed in Section 2.2.1, we will formulate the PCCMM matrix and the measurement process that maps formula vectors to PCCMM set vectors, that is, the forward propagation in the CMM–PCCMM network.

As subsets of I and J , the TCM formulas and PCCMM sets are encoded as 0–1 vectors, which are denoted as $x\in {\left\{0,1\right\}}^{N}$ (the formula vector) and $y\in {\left\{0,1\right\}}^{m}$ (the PCCMM set vector), respectively. Notably, any CMM combination, as a subset of I , can be encoded as 0–1 vectors in ${\left\{0,1\right\}}^{N}$ , but, in general, it does not necessarily form a TCM formula due to the lack of pharmacological significance. We will return to this issue in Section 2.3.1. The sparse feature of the formula vector is straightforward. In particular, supp(x) provides the indices of all the CMMs in space I that build the formula. For example, if a TCM formula has five different CMMs in I , the corresponding formula vector x is 5-sparse, that is, $\|x{\|}_{0}=\|x{\|}_{1}=5$ . To model the map from the formula vectors to PCCMM set vectors, we define the following PCCMM matrix.

Definition 1 (PCCMM matrix) *Let* $I=\left\{a_{1},a_{2},\ldots ,a_{N}\right\}$ and $J=\left\{b_{1},b_{2},\ldots ,b_{m}\right\}$ be the space of CMMs and PCCMMs, respectively. The PCCMM matrix P is a Boolean matrix of the form

$$P=\left[p_{1},p_{2},\ldots ,p_{N}\right]\in {\left\{0,1\right\}}^{m\times N},$$

where $p_{i}\in {\left\{0,1\right\}}^{m}$ , as a column vector of the matrix P , is the PCCMM set vector corresponding to the CMM $a_{i}$ in I , and i=1,2…,N .

Notably, the order of CMMs and PCCMMs in the set I and J does not affect the CMM–PCCMM networks. In terms of the PCCMM matrix P , according to definition 1, rearranging the elements in I or J leads to column or row permutations of the PCCMM matrix, respectively. In other words, the PCCMM matrix P is unique up to permutations.

With the PCCMM matrix P , we can model the map from the formula vectors to PCCMM set vectors as a quasi-linear measurement process,

$$y=\min\left\{Px,\;1\right\},\;x\in {\left\{0,1\right\}}^{N},$$ (4)

where min{⋅,1} is defined based on the entries. Like the HPE-GCN model (Liu et al., 2022), we measure the PCCMMs of a TCM formula in Equation 4 as the union of the PCCMMs of all CMMs within the formula. In other words, Equation 4 defines a qualitative PCCMM measurement that ignores the complex interactions between different CMMs. Such simplification allows us to focus on the significance of PCCMM as a representation of the TCM formula’s medication pattern from the perspective of feature extraction. We will return to the issue in Section 4.

Given a formula vector x , Px counts the number of times each PCCMM appears among the CMMs in the formula, and the “ min ” operation in Equation 4 disregards the possible multiple appearances of PCCMMs. As a result, y in Equation 4 is less informative than the linear measurement.

$$y_{w}=Px,\;x\in {\left\{0,1\right\}}^{N}.$$ (5)

As $y_{w}$ counts the multiplicities of PCCMM, we call $y_{w}$ the weighted PCCMM set vector. By interpreting y and $y_{w}$ as observed data associated with a TCM formula, we will consider the sparse recovery problem introduced in Section 2.2.1. In Section 2.2.3, we will formulate the sparse recovery problem into combinatorial optimization problems and study the capabilities of the two measurements in recovering formula vectors.

To facilitate understanding, we use Mahuang Decoction (ACFP-1-4, see Supplementary Table S4 for the details) as an example. The formula contains four CMMs: *Ephedrae Herba* (Mahuang, CMM-307), *Cinnamomi Ramulus* (Guizhi, CMM-173), *Glycyrrhizae Radix et Rhizoma* (Gancao, CMM-144), and *Armeniacae Semen Amarum* (Kuxingren, CMM-269). Thus, the corresponding formula vector x is a 0–1 vector that satisfies

$$supp\left(x\right)=\left\{144,\;173,\;269,\;307\right\}.$$

Following Equation 4 and Equation 5, we compute the PCCMM set vectors, y , and weighted PCCMM set vectors, $y_{w}$ , of Mahuang Decoction, respectively. According to their definitions, the two vectors share the same support. In particular, we have

$$supp\left(y\right)=supp\left(y_{w}\right)=\left\{1,\;51,\;52,\;62,\;65,\;74,\;76,\;86,\;112,\;113,\;124,\;139,\;141\right\},$$

which uniquely determines the 0–1 vector y . Whereas $y_{w}$ counts the multiplicities of the PCCMM appearance, we have PCCMM-51 appearing twice (CMM-307 and CMM-269), PCCMM-52 appearing twice (CMM-307 and CMM-173), and PCCMM-74 appearing twice (CMM-307 and CMM-173). Thus, the 51st, 52nd, and 74th components of $y_{w}$ are 2, rather than 1 at the corresponding position in y . The example illustrates that the weighted PCCMM set vectors usually contain more information than the unweighted ones. Table 2 summarizes the mathematical notations introduced in this section, which we will consistently use throughout the paper.

**TABLE 2:** List of frequently used mathematical notations introduced in Section 2.2.2.

x	Formula vector
y	PCCMM set vector
$y_{w}$	Weighted PCCMM set vector
P	PCCMM matrix

#### 2.2.3 Constructing the combinatorial formula model based on PCCMM

In this section, we characterize the backward propagation of the CMM–PCCMM network as a sparse recovery problem and propose a constrained combinatorial optimization model to solve it. The sparse recovery problem involves reconstructing a TCM formula from its PCCMM set. Given a target PCCMM vector $y^{†}$ corresponding to an unknown underlying formula vector $x^{†}$ , that is, $y^{†}=\min\left\{Px^{†},1\right\}$ , our objective is to recover the underlying formula vector $x^{†}$ . As our goal is similar to the spare recovery problem in compressive sensing, inspired by equation (Equation 3), we will try to identify $x^{†}$ *via* a constrained $\ell_{1}$ -optimization problem.

Let $z\in {\left\{0,1\right\}}^{N}$ denote the candidate formula vector. Then, z is associated with $y^{†}$ in the sense that $y^{†}=\min\left\{Pz,1\right\}$ . In practice, we consider its linear relaxation $Pz\geq y^{†}$ . In addition, we denote the incompatible CMM pairs (indices) as $Q=\left\{\left(i_{1},i_{2}\right),\left(i_{3},i_{4}\right),\ldots ,\left(i_{2q-1},i_{2q}\right)\right\}$ , where each pair $\left(i_{2k-1},i_{2k}\right)$ indicates that $a_{i_{2k-1}}$ and $a_{i_{2k}}$ in the CMM space I cannot appear simultaneously in a TCM formula.

To address the issue caused by relaxation, we added an $\ell_{1}$ -penalty term $\|\min\left\{Pz,1\right\}-y^{†}{\|}_{1}$ to the lost function in Equation 3 and reached the following combinatorial optimization problem.

$$\underset{z\in {\left\{0,1\right\}}^{N}}{\min}\omega_{1}\|z{\|}_{1}+\left(1-\omega_{1}\right)\|\min\left\{Pz,1\right\}-y^{†}{\|}_{1}.$$ (6)

$$s.t.\;Pz\geq y^{†},\;z_{i_{2k-1}}+z_{i_{2k}}\leq 1,\;k=1,2,\ldots ,q.$$ (7)

In the lost function (Equation 6), the $\ell_{1}$ -norm of z measures the sparsity of the candidate formula vector, and the penalty term $\|\min\left\{Pz,1\right\}-y^{†}{\|}_{1}$ quantifies the total amount of extra PCCMMs introduced by the candidate formula. Intuitively, the minimizing process aims to accomplish two goals: using the fewest kinds of CMMs to build the formula and controlling the amount of off-target PCCMMs. The hyperparameter $\omega_{1}\in \left(0,1\right)$ balances the two objectives. In Section 3.1.2, we will discuss how the value of $\omega_{1}$ affects the performance in recovering $x^{†}$ together with empirical guidance on the value selection. Unlike continuous problems such as Equation 3, Equations 6, 7 belong to the combinatorial optimization category as the decision variable z is an integer vector due to its pharmacological significance. Here, the combinatorial feature primarily influences the choice of the solver rather than the problem formulation. The numerical details are provided in Section 2.2.4.

Alternatively, we also consider the case where the available measurement is the weighted PCCMM set vector, denoted by $y_{w}^{†}$ , satisfying $y_{w}^{†}=Px^{†}$ . The corresponding combinatorial optimization problem is given as follows:

$$\underset{z\in {\left\{0,1\right\}}^{N}}{\min}\omega_{1}\|z{\|}_{1}+\left(1-\omega_{1}\right)\|Pz-y_{w}^{†}{\|}_{1}.$$ (8)

$$s.t.\;Pz\geq y_{w}^{†},\;z_{i_{2k-1}}+z_{i_{2k}}\leq 1,\;k=1,2,\ldots ,q.$$ (9)

It is worth mentioning that we can extend the combinatorial optimization model to quantitative cases by considering continuous decision variables. However, at this point, we focus on reconstructing ACFPs utilizing qualitative PCCMM data, and we shall not extend our inquiry to encompass the quantitative dimensions of the subject matter.

#### 2.2.4 Rebuilding the ACFPs from PCCMM

Based on the inverse problem of rebuilding the TCM formula from its PCCMM information and the corresponding constrained combinatorial optimization problems (Equations 6, 7 and Equations 8, 9) in Sections 2.2.3, we will present the numerical experiments and evaluation methods in this section.

The ACFP dataset consists of 178 sample test problems, and each problem is identified by vectors, namely, $x^{†}$ (formula vector), $y^{†}$ (PCCMM set vector), and $y_{w}^{†}$ (weighted PCCMM set vector). Here, the formula vector, $x^{†}$ , is encoded from CMMs in the prescriptions, whereas $y^{†}$ and $y_{w}^{†}$ are computed by measurement processes (Equations 4, 5), respectively, based on the PCCMM matrix.

To evaluate the level of restoration of ACFPs, we used the Jaccard similarity coefficient (Zeng et al., 2019) to measure the similarity between the estimated formula and the underlying true ACFPs. Recalling that, the Jaccard similarity coefficient of two non-empty sets $S_{1}$ and $S_{2}$ , denoted by $Jaccard\left(S_{1},S_{2}\right)$ , is defined as

$$Jaccard\left(S_{1},S_{2}\right)=\frac{card\left(S_{1}\cap S_{2}\right)}{card\left(S_{1}\cup S_{2}\right)}.$$ (10)

As the formulas are encoded as 0–1 vectors, let $\hat{x}$ be an estimate of the true formula vector $x^{†}$ , and we extend the definition of the Jaccard similarity coefficient (Equation 10) to

$$Jaccard\left(x^{†},\hat{x}\right)=\frac{\|\min\left\{x^{†},\hat{x}\right\}{\|}_{1}}{\|\max\left\{x^{†},\hat{x}\right\}{\|}_{1}},$$ (11)

where the “ min ” and “ max ” operations, similar to (4), are performed based on the entry. For each sample problem, estimates $\hat{x}$ are the solutions of constrained combinatorial optimization problems (Equations 6, 7 and Equations 8, 9) and are subject to the input PCCMM set vector and the weighted PCCMM set vector, respectively. We selected the Jaccard similarity coefficient (Equation 11) as the metric to quantify the level of restoration of ACFPs as the formulas are encoded as 0–1 vectors, focusing on the presence of CMMs.

Numerically, we implemented Gurobi Optimizer 10.0.2 (Gurobi Optimization, LLC, 2023) with Python 3.8.4 on an Intel™ Core i5-1135G7 2.40 GHz CPU. In our optimization process, we set poolgap=0 to prevent the accumulation of the solution pool during the computation. We set poolsolutionnum=15,000 to specify the maximum number of solutions stored in the solution pool during the optimization process. As for the hyperparameter $\omega_{1}$ in the lost functions (Equations 6, 8), a common choice is $\omega_{1}=0.5$ (Pang et al., 2014). To further explore how the value of $\omega_{1}$ affects the model performance, we incrementally set $\omega_{1}$ from 0.1 to 0.9 in steps of 0.1. Detailed numerical results are reported in Section 3.1.2.

### 2.3 Differentiation of the ACFPs based on PCCMM

In this section, we will return to the measurement process (5) and aim to explore the potential of PCCMMs in classifying ACFPs.

#### 2.3.1 Separating ACFPs from random pseudo-formulas

In Section 2.2.4, we have introduced two ACFP datasets of 93 and 85 formulas (Table 1), respectively. As representatives of TCM formulas, they should be distinguished from arbitrary CMM combinations. Therefore, in this section, we consider the clustering problem between the ACFP datasets and random pseudo-formulas based on their PCCMM set vectors. We want to explore whether the PCCMMs can separate ACFPs from randomly generated formulas of no pharmacological significance.

We use the Bernoulli’s trails to help generate the random formula dataset of desirable sparsity. Recalling that a Bernoulli random variable with parameter p , denoted as $B_{p}$ , satisfies the probability density,

$$P\left(B_{p}=1\right)=p,\;P\left(B_{p}=0\right)=1-p.$$

Let $X=\left(X_{1},X_{2},\ldots ,X_{N}\right)$ be an N -dimensional 0–1 random variable where $\left\{X_{i}\right\}$ are the i.i.d. copies of $B_{p}$ . Then, we have

$$E\left[\|X{\|}_{1}\right]=\overset{N}{\underset{i=1}{\sum }}E\left[\left|X_{i}\right|\right]=NE\left[B_{p}\right]=Np.$$

Thus, given a target sparsity s∈(0,N) , by taking p=s/N , X is an N -dimensional 0–1 random variable that satisfies $E\left[\|X{\|}_{1}\right]=s$ . Moreover, as $\|X{\|}_{1}$ is the sum of N i.i.d. Bernoulli random variables, $\|X{\|}_{1}$ follows the binomial distribution. In our clustering problem, we sampled the vector $x\in {\left\{0,1\right\}}^{N}$ from X with s=6.55 (the average number of CMMs per prescription over the two batches of ACFP datasets in Table 1) to generate the pseudo-formula dataset.

We mixed the three formula vector datasets and the corresponding weighted PCCMM set vector datasets computed by Equation 5. In other words, we contaminated the ACFP dataset with irrelevant information from the pseudo-formula. As high-dimensional vectors, it is difficult to tell directly whether the point clouds formed by the two types of vectors yield clusters or not under the unsupervised learning setup. As a remedy, we employed t-distributed stochastic neighbor embedding (t-SNE) (Pezzotti et al., 2017) as the dimensional reduction method, which helped us picture the potential clusters. In manifold learning, t-SNE aims to represent high-dimensional points in lower dimensions while preserving their similarities. The t-SNE algorithm finds the similarity measure between pairs of instances in higher and lower dimensional spaces and tries to optimize two similarity measures in the following three steps (Bo et al., 2021):

(i) t-SNE models a point selected as a neighbor of another point in both higher and lower dimensions. It starts by calculating a pairwise similarity between all data points in the high-dimensional space using Gaussian kernels. The points that are far apart have a lower probability of being picked than the points that are close together.
(ii) Then, the algorithm tries to map higher dimensional data points onto lower dimensional space while preserving the pairwise similarities.
(iii) It is achieved by minimizing the divergence between the probability distribution of the original high-dimensional and lower dimensional space. The algorithm uses gradient descent to minimize the divergence. The lower dimensional embedding is optimized to a stable state.

We used MATLAB 2021a simulation software for data preparation and 2D t-SNE implementation. In the “tsne” input arguments, we selected the hamming distance as the distance function in the t-SNE. Recalling that the hamming distance between two vectors corresponds to the number of inconsistent entries, we set LearnRate=500 and Perplexity=30 to optimize the visualization.

#### 2.3.2 Distinguishing the deficiency syndromes of the ACFPs

Here, we merged the two batches of ACFPs into a single dataset and considered the clustering problem based on the syndromes and efficacy of the prescriptions.

The syndromes and efficacy of the prescriptions were retrieved through the China National Knowledge Infrastructure (CNKI), PubMed, Web of Science, and other databases. In this study, we selected the prescriptions of deficiency syndromes and classified them under the four labels of Yang deficiency pattern (23 cases), Yin deficiency pattern (24 cases), Qi deficiency pattern (27 cases), and blood deficiency pattern (21 cases) (World Health Organization, 2022), and introduced the supervised learning problem by splitting data into the training and testing sets.

Due to the limited data available, we utilized the bootstrap method. In statistics, the bootstrap method is a resampling technique that involves repeatedly sampling with replacement from the original data to estimate the distribution of parameters and calculate confidence intervals (Efron, 1979). For the deficiency pattern clustering problem, instead of fixing the training sets, we repeatedly resampled the prescriptions to build the training data and solved the supervised learning problem. We summarized the learning process as follows:

1) As for data preprocessing, we used the 2D t-SNE, subject to hamming distance, LearnRate=500 and Perplexity=30 . 2) For each deficiency pattern, we uniformly selected 16 cases (with labels) to build the training set. The rest of the cases (without labels) formed the test set. 3) For each sample in the test set, we computed its $\ell_{1}$ -distance to the cases in the training set. Then, we assigned the formula to the deficiency pattern of the shortest average distance as the prediction. (iv) We compared the predicted labels with the true deficiency pattern and collected the correctness.

We set the number of trials in the bootstrap method to 5,000 and applied the above learning process to both formula vectors and weighted PCCMM set vectors. The detailed numerical results are reported in Section 3.2.2.

### 2.4 Medication pattern analysis of the ACFPs based on PCCMM

Based on CMM and PCCMM, we explored the medication patterns of ACFPs. The “itemFrequency” function of R 4.3.2 was used for frequency analysis. On this basis, association rule analysis, correlation analysis, and cluster analysis on high-frequency CMM and PCCMM were performed, respectively, based on the “*a priori*” function, “corrplot” function, and “hclust” function, and the above results were visualized.

## 3 Results

### 3.1 Evaluating the feature extraction of the ACFPs

#### 3.1.1 The evaluation of the PCCMM matrix

**FIGURE 2:**
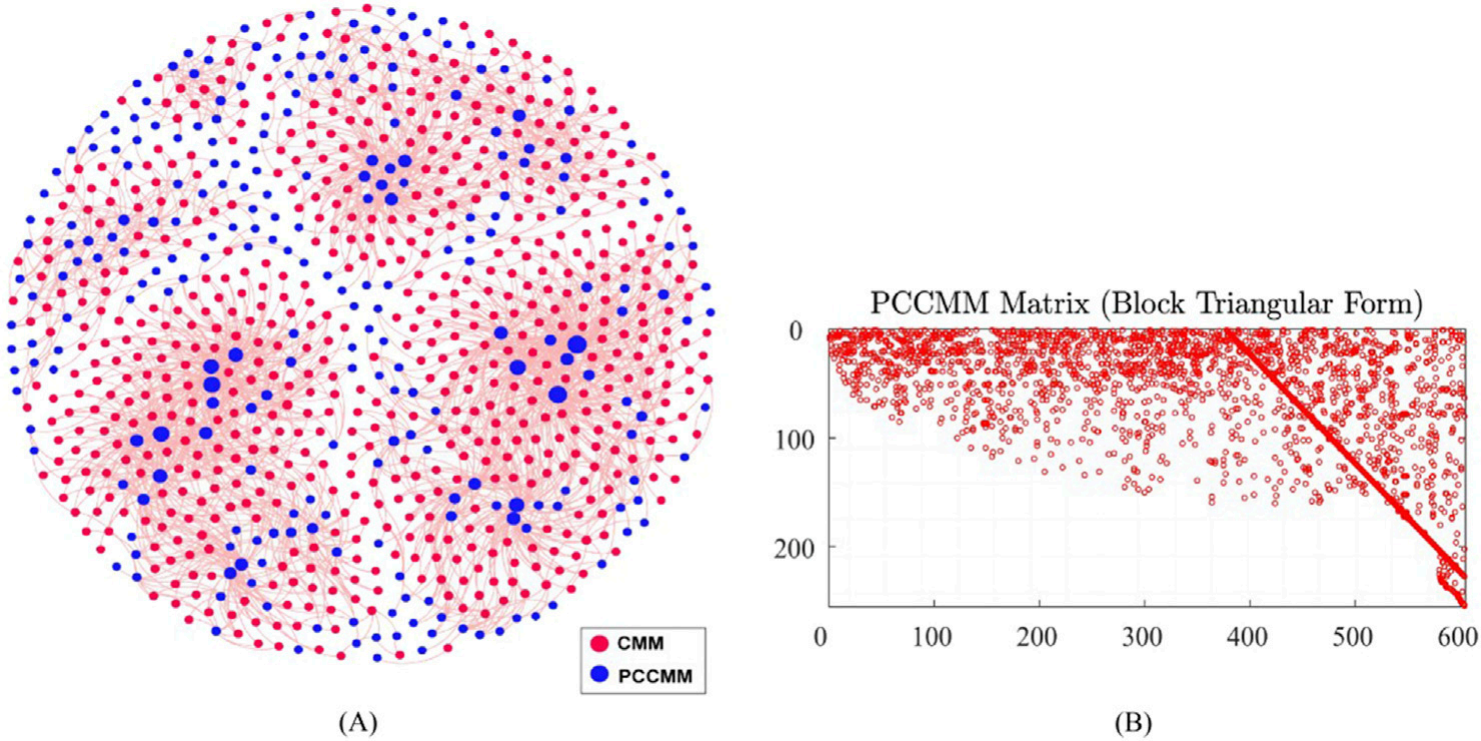
Visualizing the CMM–PCCMM network and the sparsity pattern of the PCCMM matrix P . **(A)** CMM–PCCMM network. CMM nodes are in red, whereas PCCMM nodes are in blue. The size of the PCCMM nodes is directly proportional to its degrees, meaning the larger the blue circle, the more frequently that particular PCCMM occurs. **(B)** Sparsity pattern of the PCCMM matrix P . The horizontal axis represents the column index of the matrix, ranging from 1 to 604; the vertical axis represents the row index of the matrix, ranging from 1 to 254. A 254×604 Boolean matrix of 2216 nonzero elements. Nonzero values are in red, whereas zero values are in white. We used the MATLAB function “dmperm” to obtain the block triangular form.

Based on the 604 CMMs and their corresponding PCCMMs introduced in Section 2.1.1, we constructed the CMM–PCCMM network and visualized this binary network in Figure 2A using Gephi version 0.9.2 (Bastian et al., 2009). The CMM–PCCMM network encompasses 604 CMM nodes, 254 PCCMM nodes, and 2,216 edges.

To evaluate the PCCMM matrix P defined by definition 1, we transformed the CMM–PCCMM network into a PCCMM matrix of size 254×604 , and there are a total of 2,216 nonzero elements in the PCCMM matrix. We found that the PCCMM matrix is sparse (less than 1.5% of nonzero elements). To visualize the sparsity pattern of the PCCMM matrix, we applied row and column permutations to P and obtained its block triangularization form that is displayed in Figure 2B.

#### 3.1.2 The performance of rebuilding ACFPs from the PCCMM

**FIGURE 3:**
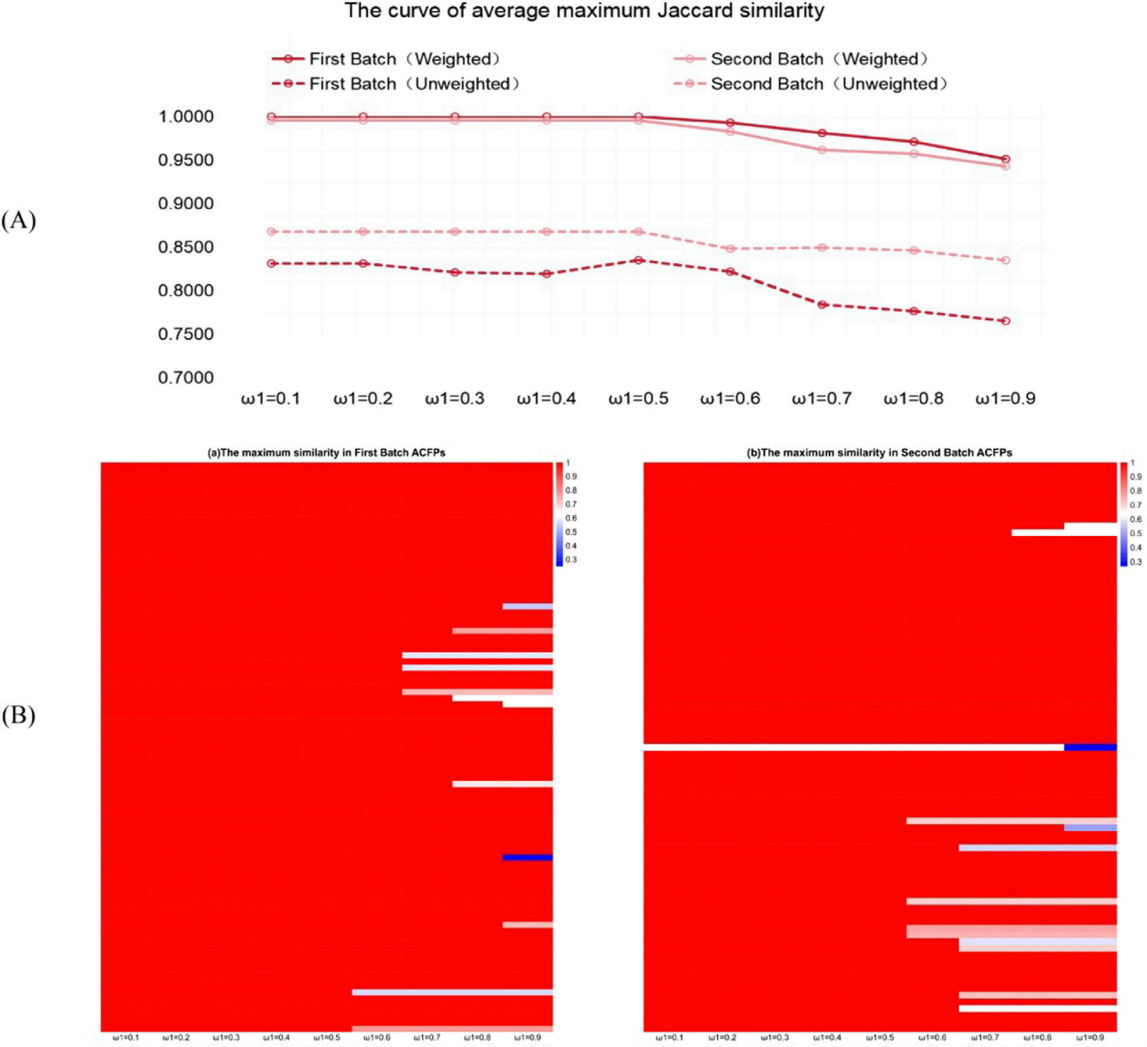
Jaccard similarity of rebuilding ACFPs from the PCCMM. **(A)** Curve of the average maximum Jaccard similarity as a function of the hyperparameters $\omega_{1}$ . We varied the value of $\omega_{1}$ from 0.1 to 0.9 with a step size 0.1. The solid and dashed lines correspond to the weighted and unweighted PCCMM set vectors, respectively. The two batches have similar trends. After $\omega_{1}\geq 0.5$ , the average maximum Jaccard similarities decay as $\omega_{1}$ increases. **(B)** Heatmap of the maximum value among the Jaccard similarity coefficients between the estimated CMM combinations (based on weighted PCCMM set vector) and ACFPs in the first batch (the left panel) and second batch (the right panel). The x -axis corresponds to the value of $\omega_{1}$ . We used Hiplot (Li J. et al., 2022) to prepare the heatmap.

As reported in Table 1, we considered 178 prescriptions from the first and second batches of the ACFP catalogs (Supplementary Tables S4, S5) to form the sample problem dataset. For each sample problem, we inputted the PCCMM set vector $y^{†}$ and the weighted PCCMM set vector $y_{w}^{†}$ to the combinatorial optimization problems (Equations 6–9), respectively. As, in general, the estimated solutions produced by the solver are not unique, under each hyperparameter value, we collected the multiple optimal solutions as the solution pool and calculated their Jaccard similarity coefficient (Equation 11) with respect to the underlying true formula vector $x^{†}$ . As we were interested in the capability of PCCMM to recover the ACFPs, we reported the Jaccard similarity among the estimates generated by the solver (Figure 3).

From Figure 3A, we can see that the weighted PCCMM set vector $y_{w}^{†}$ performs better. This is unsurprising, as it contains richer PCCMM information by counting the multiplicities of the PCCMM occurrence in the formula. As for the hyperparameter $\omega_{1}$ , when $\omega_{1}\geq 0.5$ , the performance in reconstructing ACFPs gradually declines in the two batches as $\omega_{1}$ increases. In the lost function (Equations 6, 8), as $\omega_{1}$ approaches 1, the $\ell_{1}$ -penalty terms become less and less influential in the optimization problem, which leads to an increasing amount of off-target PCCMMs in the estimates and a drop of the recovering performance. The curves support $\omega_{1}=0.5$ as the hyperparameter value, which is consistent with the conventional choice used in the study by Pang et al. (2014). Under $\omega_{1}=0.5$ , all the averaged values of the Jaccard similarity coefficients exceed 0.8. The positive performance in rebuilding ACFPs from the PCCMM information demonstrates the well-posedness of the backward propagation in the CMM–PCCMM network introduced in Section 2.2.3. In other words, the PCCMM measurement, especially the weighted PCCMM set vectors, can be interpreted as quasi-equivalent representations of the ACFPs, and analyzing the PCCMM information is an effective tool for understanding the TCM formula.

The heatmap in Figure 3B reports the individual recovering performance of each sample problem based on the weighted PCCMM set vector as $\omega_{1}$ varied. Only the prescription of ACFP-2–46 (LiuHe Decoction) was not reconstructed throughout the process. For this sample problem, when $\omega_{1}=0.1$ , the four estimated formula vectors in the solution pool reached the maximum similarity of 0.615 listed in Table 3. After investigating the solution pool under $\omega_{1}=0.1$ , we found that all 128 solutions met the constraint in Equation 9, [Disp-formula e8]. Moreover, the estimates use fewer CMMs (10 CMMs) to achieve the target PCCMM than the original prescription (11 CMMs).

**TABLE 3:** Original prescription of ACFP-2–46 and the four estimated formulas of the largest Jaccard similarity and fewer CMMs.

TCM-formula	CMM code^a^	Jaccard
Original	CMM-11, CMM-32, CMM-139, CMM-169, CMM-200, CMM-269, CMM-335, CMM-385, CMM-405, CMM-496, and CMM-558	–
Estimate 1	CMM-32, CMM-114, CMM-139, CMM-145, CMM-200, CMM-269, CMM-335, CMM-385, CMM-496, and CMM-558	0.615
Estimate 2	CMM-32, CMM-139, CMM-145, CMM-200, CMM-269, CMM-329, CMM-335, CMM-385, CMM-496, and CMM-558	0.615
Estimate 3	CMM-139, CMM-145, CMM-169, CMM-200, CMM-269, CMM-329, CMM-335, CMM-385, CMM-496, and CMM-558	0.615
Estimate 4	CMM-114, CMM-139, CMM-145, CMM-169, CMM-200, CMM-269, CMM-335, CMM-385, CMM-496, and CMM-558	0.615

^a^ All four estimated formulas comprise 10 CMMs, whereas the original prescription contains 11 CMMs. The estimates’ CMM codes that overlap with the CMM code of the original prescription are displayed in bold font.

To explain why there are four estimated formulas of the same Jaccard similarity, we checked the PCCMMs of the CMM that distinguish these estimates. We found that both CMM-32 (Pinelliae Rhizoma, Banxia) and CMM-169 (Pogostemonis Herba, Guanghuoxiang) belong to the following PCCMMs: warm–pungent–lung meridian, warm–pungent–spleen meridian, and warm–pungent–stomach meridian. Furthermore, the two CMMs have the ability to alleviate dampness and prevent vomiting. In addition, CMM-114 (Caryophylli Flos, Dingxiang) and CMM-329 (Caryophylli Fructus, Mudingxiang) also share the same PCCMMs: warm–pungent–lung meridian, warm–pungent–spleen meridian, warm–pungent–kidney meridian, and warm–pungent–stomach meridian, and they have the same efficacy and indications in ChP. Therefore, the PCCMM measurement cannot distinguish between CMM-32 and CMM-169, as well as between CMM-114 and CMM-329, which elucidates the four estimates presented in Table 3.

Furthermore, the numerical experiments indicate that the results based on the ChP 2015 edition are consistent with those derived from the 2020 edition, exhibiting the same maximal Jaccard similarity values and underscoring the robustness of this approach across different reference databases. To further validate the adequacy of Jaccard similarity in our numerical experiment, we reported the comparison between cosine and Jaccard similarities in Supplementary Figure S1, which reveals a high consistency in trends between two similarity curves.

### 3.2 Distinguishing the ACFPs from different dimensions

#### 3.2.1 The performance of separating ACFPs from random pseudo-formulas

To form the CMM/PCCMM datasets used for the clustering problem, following Section 2.3.1, we generated 150 random pseudo-formula vectors and computed their weighted PCCMM set vectors *via* Equation 5, [Disp-formula e8]. Mixing them with the 178 ACFPs in the two batches in Table 1, we formed the CMM/PCCMM dataset, each consisting of 328 vectors. In this section and also in Section 3.2.2, motivated by the result in Section 3.1.2, we used the weighted PCCMM set vectors to build the PCCMM dataset.

**FIGURE 4:**
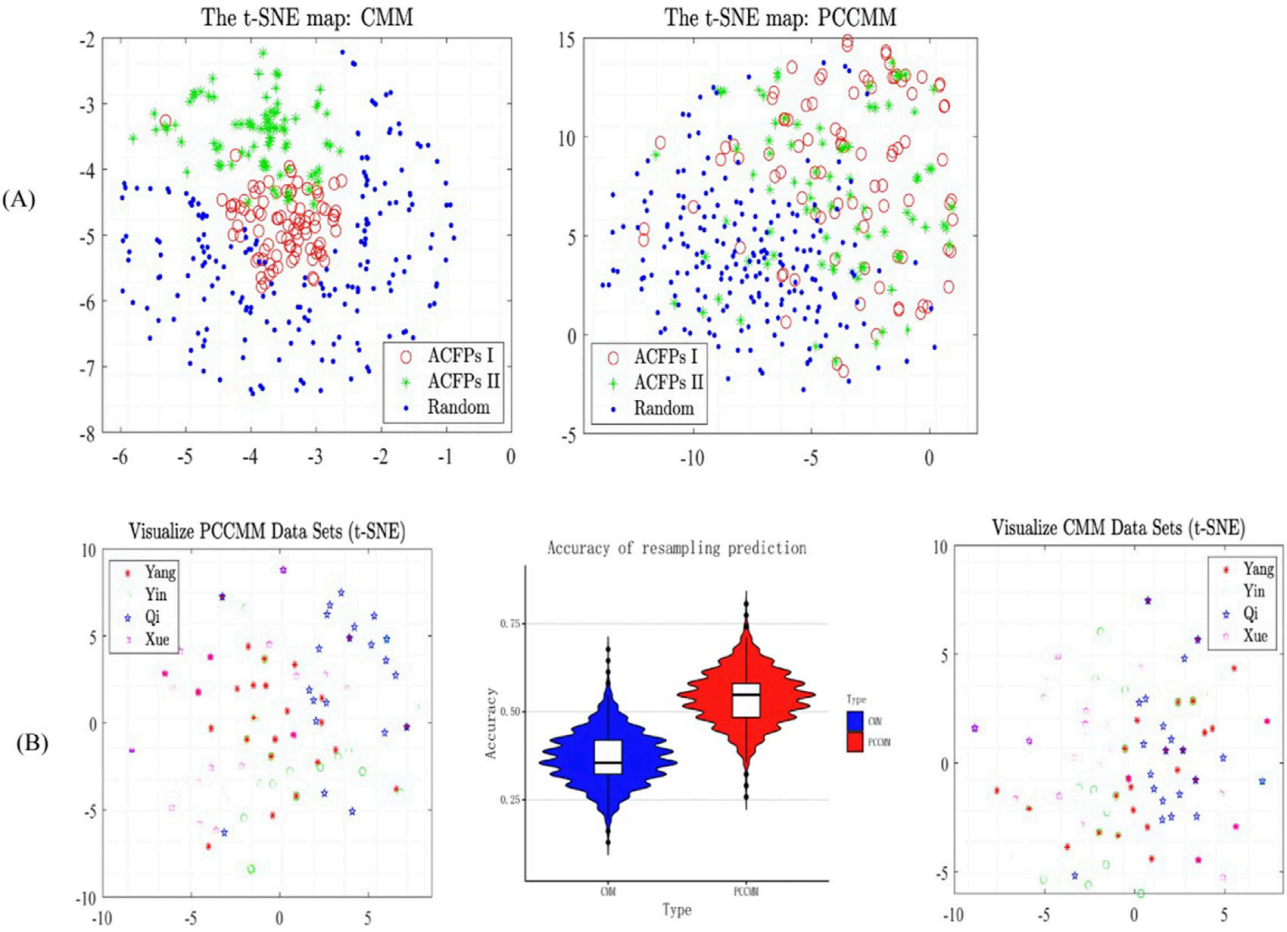
Differentiating the ACFPs based on PCCMM and CMM. **(A)** Scatterplots of the t-SNE maps of the formula vectors (the left panel) and weighted PCCMM set vectors (the right panel). In the scatterplots, each data point was assigned with the original labels. “ACFPs I” and “ACFPs II” stand for the ACFPs in the first and second batches, respectively. “Random” corresponds to the random pseudo-formulas. The cluster information (“ACFPs I,” “ACFPs II,” and “Random”) of each vector is only used in the scatterplot plotting. In the right panel, there is an approximately linear boundary between the clusters of ACFPs and random pseudo-formulas. Additionally, the distribution patterns of the weighted PCCMM vectors in the two batches of ACFPs are similar, which is different from the patterns of the CMM vectors in the left panel. **(B)** Classification of the four deficiency syndromes in ACFPs based on CMM and PCCMM. The left and right panels report the t-SNE map of the four deficiency syndromes in ACFPs based on CMM and PCCMM, respectively. The middle panel displays a violin plot of the bootstrap method’s correct rate distribution and quantiles, with the plot’s wiggles reflecting the discrete distribution of the correct rate. The violin plot shows that PCCMM has a higher correct rate than CMM in the classification tasks.

Then, we utilized the t-SNE algorithm to generate 2D projections of the CMM and PCCMM datasets, respectively, providing visual representations of the data distribution and employing Euclidean distances to measure the proximity between data points in the expression space (Bushati et al., 2011) and plotted the scatterplot (Figure 4A). The scatterplots suggest that both the CMM and PCCMM measurements can separate the ACFPs from the random pseudo-formulas, but the weighted PCCMM set vector, after being projected using t-SNE, generated an approximately linear boundary between the clusters of ACFPs and random pseudo-formulas. Thus, in contrast to CMMs, PCCMM measurements offer a more intuitive separation between two clusters, which is convenient for implementation purposes. Another interesting observation is that the distribution patterns of the weighted PCCMM vectors between the first and second batches of ACFPs are similar. Unlike the CMMs, the PCCMM measurements, after being projected using t-SNE, cannot distinguish the ACFPs from the first and second batches. Notice that the involved CMMs of the two batches are quite different (see Table 1 for details), which may explain why the formula vector can separate ACFPs from different batches. On the other hand, the PCCMM measurement is insensitive to the scope of the CMMs, which makes it a more intrinsic indicator in seeking common features among different ACFPs.

#### 3.2.2 The classification of deficiency syndromes in ACFPs by PCCMM

We retrieved 95 prescriptions relevant to deficiency syndromes from the two batches of ACFPs (Supplementary Table S6). Among them, there are 23 cases of Yang deficiency pattern, 24 cases of Yin deficiency pattern, 27 cases of Qi deficiency pattern, and 21 cases of blood deficiency pattern.

Initially, we utilized the t-SNE algorithm for data preprocessing to obtain 2D projections. However, the distribution differences among the labels of the four deficiency syndromes were not clearly distinguishable in the resulting graph (Figure 4B), making it difficult to differentiate between them visually. To address this issue, we employed the bootstrap method for supervised learning and further analyzed the distribution by resampling with replacement from the original data. Our results showed that the classification accuracy based on the PCCMM was 0.55, which outperformed the classification accuracy of 0.42 obtained using the nomenclature of CMM. The lack of training data might cause the relatively poor performance for both the measurements.

### 3.3 Analyzing the medication patterns of ACFPs

#### 3.3.1 The frequency and association rules analysis

High-frequency CMMs are those with markedly increased usage rates in clinical practice, classic prescription, or modern research, demonstrating the integration of TCM theory with clinical application, which can be identified by their high frequencies in disease-specific formula databases, clinical guidelines, or bibliometric analyses (Wang J. et al., 2021). A common approach to identifying high-frequency CMMs is frequency analysis, which involves directly counting the occurrences of CMMs. Currently, there is no unified standard for defining high-frequency CMMs. Some studies measure total occurrences, whereas others rely on proportional frequency. For instance, Liu et al. (2024) classified CMMs with a frequency greater than 3% as high frequency, whereas Yang et al. (2024) considered those occurring more than eight times as high frequency.

Among the 196 CMMs mentioned in Table 1, 34 CMMs had a frequency of ≥10 and were classified as high frequency, accounting for 63.38% of the cumulative frequency. *Glycyrrhizae Radix et Rhizoma* (Gancao), *Angelicae Sinensis Radix* (Danggui), and *Paeoniae Radix Alba* (Baishao) were the most frequently used CMMs in ACFPs (Supplementary Table S7). In Table 4, we divided the high-frequency CMMs based on their functions (Yang et al., 2021). Tonic medicines were the most frequently used, followed by interior heat-clearing medicines and exterior heat-releasing medicines. For the 176 PCCMMs, 29 PCCMMs had a frequency of ≥50 and were classified as high frequency, accounting for 57.33% of the cumulative frequency. PCCMM-112 (sweet–even–spleen meridian), PCCMM-1 (sweet–even–lung meridian), and PCCMM-65 (sweet–even–heart meridian) were the most frequently used PCCMMs in ACFPs (Supplementary Table S8).

**TABLE 4:** Classification of high-frequency CMMs based on their functions.

Types of CMMs	Frequency	Proportion (%)
Tonic medicines	337	45.60
Interior heat-clearing medicines	106	14.34
Exterior heat-releasing medicines	99	13.40
Phlegm-transforming medicines	43	5.82
Urination-promoting and dampness-draining medicines	37	5.01
Qi-regulating medicines	33	4.47
Interior-warming medicines	27	3.65
Blood-circulating and blood stasis-resolving medicines	21	2.84
Dampness-transforming medicines	19	2.57
Purgative medicines	17	2.30

#### 3.3.2 The distance and correlation analysis

**FIGURE 5:**
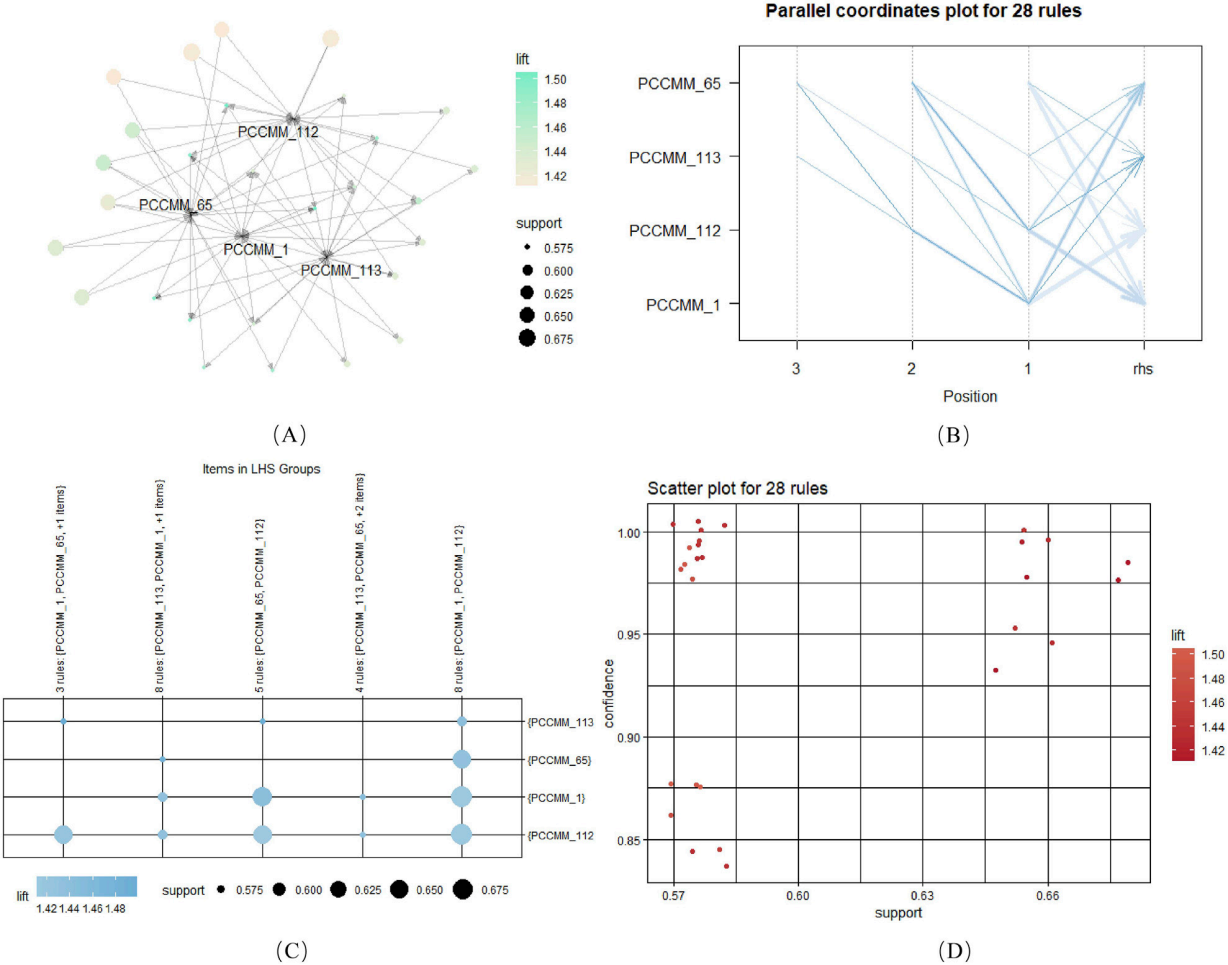
Association rules of ACFPs based on PCCMM, with a minimum support threshold of 0.5. **(A)** Network graph depicting the association rules for PCCMM-1, PCCMM-65, PCCMM-112, and PCCMM-113. **(B)** Parallel coordinate plot illustrating the 28 identified association rules. **(C)** Twenty-eight items categorized under the left-hand side (LHS) groups. **(D)** Relationship among confidence, support, and lift for the 28 rules.

The “*apriori*” function was employed to perform association rule analysis. The settings were support ≥0.1 , confidence ≥0.8 , and lift >1 . Based on CMM, no frequent item-set was obtained. However, based on PCCMM, 383,653 rules were derived (Supplementary Table S9). The advantage of PCCMM in mining implicit compatibility rules was manifested. The results with support ≥0.5 were visualized to obtain 28 frequent item-sets in Figure 5.

**FIGURE 6:**
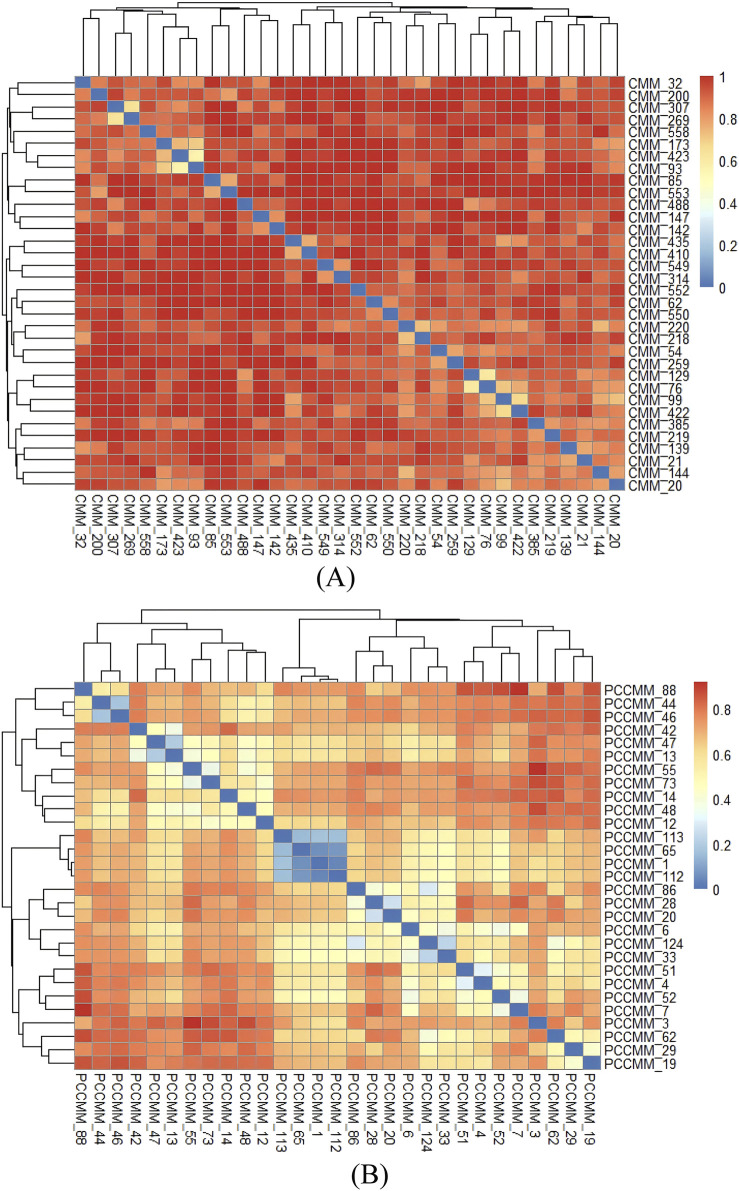
Distance of CMM pairs and PCCMM pairs of ACFPs. **(A)** Heatmap of the distance between CMM pairs. **(B)** Heatmap of the distance between PCCMM pairs. The colors of each cell represent the pairwise distance for CMM or PCCMM. Redder cells indicate larger distances, approaching 1, whereas bluer cells indicate smaller distances, approaching 0.

We calculated the pairwise distances between high-frequency CMM and high-frequency PCCMM, based on the binary distance method, and visualized them in Figure 6. The CMM pairs with the smallest distances are as follows: *Jujubae Fructus* (Dazao) and *Zingiberis Rhizoma Recens* (Shengjiang), *Saposhnikoviae Radix* (Fangfeng) and *Chuanxiong Rhizoma* (Chuanxiong), and *Angelicae Sinensis Radix* (Danggui) and *Rehmanniae Radix* (Dihuang). The PCCMM pairs with the smallest distances are as follows: even–sweet–heart meridian and even–sweet–lung meridian, even–sweet–heart meridian and even–sweet–spleen meridian, and even–sweet–lung meridian and even–sweet–spleen meridian, which indicate that they often co-occur in ACFPs, suggesting a significant synergistic effect.

Correlation analysis was performed based on the Pearson correlation coefficient (r) , and the results are visualized in Supplementary Figures S2, S3. In the figures, the CMM pair with the strongest positive correlation is *Jujubae Fructus* (Dazao) and *Zingiberis Rhizoma Recens* (Shengjiang) (r=0.55) , whereas the CMM pair with the strongest negative correlation is *Glycyrrhizae Radix et Rhizoma* (Gancao) and *Glycyrrhizae Radix et Rhizoma Praeparata Cum Melle* (Zhigancao) (r=−0.36) . The PCCMM pair with the strongest positive correlation is even–sweet–lung meridian and even–sweet–spleen meridian (r=0.93) , whereas the PCCMM pair with the strongest negative correlation is warm–pungent–lung meridian and cold–sweet–kidney meridian (r=−0.29) . From this, it can be seen that $r_{\text{max}}^{\text{PCCMM}}\left(0.93\right)>r_{\text{max}}^{\text{CMM}}\left(0.55\right)$ , indicating that stronger correlation relationships can be obtained from the PCCMM level compared to the CMM level.

## 4 Discussion

In contrast to the previous studies which primarily focused on individual prescriptions, our research systematically investigated the medication patterns of the catalog of ACFPs issued by the Chinese government. We constructed the forward and backward feature extraction processes from ACFPs to PCMM, and analyzed the medication patterns within ACFPs from the PCMM. Motivated by the sparsity feature of the TCM formulas, we employed the compressive sensing framework to establish the CMM–PCCMM network and introduced the combinatorial optimization problem to rebuild the ACFPs from their PCCMMs. The numerical results based on the ACFP datasets demonstrated that the PCCMM set is a quasi-equivalent representation of the TCM formulas and that PCCMMs outperform the nomenclature of CMMs in ACFP classification. Furthermore, PCCMM may facilitate the extraction of implicit compatibility patterns within ACFPs more effectively than CMM alone. The discussion is as follows.

### 4.1 The simplification of the combinatorial formula model

Using the compressive sensing framework, we showed the ability to recover the TCM formula from its PCCMM information *via* numerical experiments over the ACFP dataset and numerically demonstrated the legitimacy of the CMM–PCCMM network. We must point out that the quasi-linear measurement process in the combinatorial formula model is a simplified and qualitative model similar to HP-GCN (Liu et al., 2022), treating the CMMs and PCMMs as equal and independent contributors to the formula’s PCCMMs. However, the proportion of each CMM in TCM formulas varies, and previous studies have indicated significant internal correlation among the constituent CMMs in the TCM formulas (Zhang et al., 2024), suggesting important intrinsic links between PCMMs and the physicochemical properties and pharmacological actions of individual metabolites, as shown in the distribution pattern of PCMMs (Ung et al., 2007). Furthermore, among CMMs that share an identical PCMM, the potency of their PCMM is not uniform (Wei et al., 2022). As an essential basis for the composition of TCM formulas, we considered PCCMMs the minimum unit of efficacy characterization. In this study, we focus on the significance of PCCMM as a representation of the TCM formulas’ medication pattern from the perspective of feature extraction. For the sake of computational simplicity and tractability, we have adopted a simplified model that temporarily disregards the complex interactions between multiple efficacies and the variations in their effects, as well as other potential biological factors.

### 4.2 The generalizability and complexity of the sample problem set

First, the sample problem set covers more than 90% of the prescriptions in the original ACFP catalogs. Thus, our CMM–PCCMM network proposed in Section 2.2.2 is general enough for analyzing ACFPs. From Table 1, we know the average number of CMMs per prescription is 7.04 and 6.01 in the first and second batches, respectively. In other words, the average percentage of nonzero elements in the formula vector $x^{†}$ is approximately 1% . The sparsity feature of the ACFPs in the catalogs is consistent with the assumptions of the compressive sensing framework reviewed in Section 2.2.1. Notice that the number of unique CMMs involved is much less than the size of the CMM space I (N=604) used for the problem. In practice, to improve the performance and computational efficiency, one can narrow down the scope of the CMM space. Taking the first batch as an example, we can restrict the CMM space I to its subset consisting of the 155 unique CMMs. As a result, we reduce the dimension of the formula vector from 604 to 155, which significantly diminishes the difficulty of the rebuilding problem. However, we do not assume any *a priori* condition about the CMM domain and stick to the entire CMM space I in testing the generalizability of our problem.

### 4.3 The influence across ethnic groups on the research framework

According to PTCMM, the herb properties of TCM include cold, hot, warm, cool, and even. In Tibetan medicine (Dangzhi et al., 2019), the properties are cold, hot, heavy, light, dull, sharp, moist, and rough. In Ayurveda medicine (Rastogi and Singh, 2021), the properties include heavy, greasy, cold, dull, light, rough, hot, and sharp. Similarly, in Mongolian medicine (Siqi et al., 2024), the properties include hot, cold, non-oily, heavy, light, sharp, viscous, and non-viscous. Despite subtle differences, all these medical traditions recognize cold and hot as fundamental properties of botanical drugs. The herb flavors in TCM encompass sour, bitter, sweet, pungent, salty, astringent, and bland. In contrast, Tibetan, Ayurveda, and Mongolian medicine do not include bland among their recognized flavors. Furthermore, multiple medical systems, extending beyond PCMM, consistently assert that the medicinal properties of botanical drugs are inextricably linked to their therapeutic efficacy. We must point out that although the medicinal properties of botanical drugs vary across different ethnic groups, their data structures are highly similar. These properties can be uniformly represented in a multi-tuple format, which is analogous to the PCCMM framework. This consistency allows for the construction of bipartite networks, making it feasible to apply the theoretical framework developed in this study for further analysis.

### 4.4 The universality of the constructed mathematical framework

The mathematical framework of the CMM–PCCMM network presented in our paper is general, allowing for the substitution of PCCMM with other linear or quasi-linear TCM measurement formulas. It is noteworthy that in another manuscript currently under review, we have applied the feature extraction-based framework developed in this study to identify core CMM pairs for alcoholic liver disease (ALD) and to analyze key active metabolites as well as their biological mechanisms in treating ALD. In that study, we constructed a CMM–target network based on HERB (Fang et al., 2020) and HIT2.0 (Yan et al., 2021). Through bioinformatics analysis, we established a protein–protein interaction network and identified hub genes associated with ALD. Based on clinically validated TCM formulas for ALD, the data mining approach combined with combinatorial optimization modeling identified the core CMM pair of Gardeniae Fructus (Zhizi) and Artemisiae Scopariae Herba (Yinchen). Previous experimental research (Tan et al., 2023) has confirmed the hepatoprotective properties of these botanical drugs. Furthermore, the model predicted that the biological mechanism of the core CMM pair may primarily involve the antioxidant and anti-inflammatory properties of quercetin, mediated by its inhibition of COL1A1 and COL3A1 expression. Molecular docking and molecular dynamics simulations demonstrated stable binding between quercetin and both COL1A1 and COL3A1, with strong binding energy and affinity. In this study, we further validate the versatility of the framework developed in the current research and provide novel insights to advance modern TCM research and development. In addition, one could examine other pharmacopoeias, such as the United States Pharmacopeia and National Formulary, to analyze the corresponding medication patterns using the mathematical framework outlined in the paper.

### 4.5 Advantages and prospects

Our work has the following significance:

(i) Providing a basis for the exploration of implicit compatibility patterns in TCM formulas based on PCCMM: unlike traditional methods that rely on CMMs, our approach introduces PCCMM as a novel approach for mining medication patterns of ACFPs. Our work enhances the capability of PCCMM for serving as a quasi-equivalent representation of TCM formulas. The classification and identification of ACFPs using PCCMM information yield superior results compared to using CMM information alone, providing a basis for the exploration of implicit compatibility patterns in TCM formulas based on PCCMM. Moreover, compared with CMMs, the PCCMM measurement is more applicable for research related to TCM formula datasets (rather than an individual prescription), for example, clustering TCM formulas and identifying shared features between different TCM formulas.
(ii) Exploring the implicit compatibility patterns of ACFPs using PCCMM: we found that the PCCMMs of ACFPs predominantly map to the even–sweet–spleen meridian. The number of associate rules derived from PCCMM significantly exceeded that derived from CMM, highlighting PCCMM’s advantage in mining hidden compatibility patterns. Data mining revealed positively and negatively correlated PCCMM pairs, potentially guiding the discovery of synergistic and contraindicated CMM combinations in TCM. For instance, The CMM pair with the strongest positive correlation is *Jujubae Fructus* (Dazao) and *Zingiberis Rhizoma Recens* (Shengjiang) (r=0.55) , suggesting that this combination may exhibit synergistic effects. Through literature review, we found that both of these CMMs are simultaneously present in several TCM formulas, including Chaihu Guizhi Decoction (Zhao et al., 2024), Da-Chai-Hu-Tang Formula (Duan et al., 2024), and Zhi-Gan-Cao-Tang (Hu et al., 2023). This combination has the effects of harmonizing the defensive and nutritive qi, regulating the spleen and stomach, warming the meridians, and promoting circulation (Liao et al., 2023). Similarly, the PCCMM pair with the strongest positive correlation is even–sweet–lung meridian and even–sweet–spleen meridian (r=0.93) . For example, both Codonopsis Radix (Dangshen) and Poria (Fuling) contain this PCCMM pair. Existing pharmacological studies have demonstrated that they may exert synergistic effects in treating ALD by modulating the expression of AKT, TNF, and MAPK, thereby reducing inflammatory cell infiltration. This indicates that CMMs containing these PCCMMs may have significant synergistic effects when used in combination, and the PCCMM-based approach could facilitate the discovery of novel herb pairs with potential synergistic properties.
(iii) Providing a new perspective for designing new TCM formulas and optimizing existing TCM formulas: in this study, during the rebuilding of the ACFPs based on the combinatorial formula model, a new set of estimated values for CMMs was obtained under constrained conditions. For instance, the estimated values for CMMs derived from our model align more closely with the constraints than the original prescription (ACFP-2-46, LiuHe Decoction) and exhibit a reduced number of CMMs. A prescription with fewer CMMs yet unchanged or improved efficacy is one of the approaches to optimizing TCM formulas. Taking estimate 1 in Table 3 as an example, compared to the original ACFP, it excludes CMM-11, CMM-169, and CMM-405 while incorporating CMM-114 and CMM-145. Through a comprehensive review of *Clinical Chinese Pharmacy* (Zhang et al., 2020), we identified that the three excluded CMMs are commonly used in modern clinical practice for treating acute and chronic gastroenteritis, as well as gastrointestinal dysfunction. Notably, the two newly added CMMs exhibit similar therapeutic efficacy, particularly in managing chronic gastritis and gastrointestinal disorders. We hypothesize that this might represent an optimized version of LiuHe Decoction with a streamlined list of CMMs, potentially offering a new perspective for TCM formula optimization. Of course, further investigation in subsequent studies is necessary, where such sets of solutions should be further observed in conjunction with experts’ experience or clinical practice.
(iv) Facilitating Western medicine’s acceptance of TCM drug development: in this study, we selected ACPFs as representative samples of TCM formulas and developed a feature extraction model based on PCCMM to reveal their compatibility patterns. We utilized the Jaccard index to quantitatively assess the model’s ability to rebuild ACFPs; however, our CMM–PCCMM network does not depend on Jaccard similarity or any specific similarity measurement. We believe that incorporating quantifiable metrics like Jaccard similarity can enhance Western medical practitioners’ understanding of PTCMM and provide a clearer interpretation of CMM compatibility principles. This approach offers an objective perspective on TCM formulas’ principles based on PCMM.

### 4.6 Future works

The limited dataset used in the numerical experiments is the main drawback of our study. Both the CMM and PCCMM information are encoded as integer vectors in this study, which did not account for the impact of formula proportions on the properties of ACFPs. The impact of formula proportions on the properties of ACFPs has not been considered. Looking ahead, upon completion of the dosage verification for certain ACFPs, we will attempt to incorporate the dosage of CMMs in TCM formulas and combine it with the PCCMM measurements to refine the weighted PCCMM set vector and introduce the quantitative CMM–PCCMM network. Our long-term goal includes PCCMM-guided methods for TCM formula construction or optimization. Our study emphasizes the role of PCCMM in revealing the medication patterns of ACFPs from the perspective of systematic science. In general, the PCCMM characteristics can be determined by the substances beyond the scope of the CMM–PCCMM network, such as food, nutritional components, and chemical compositions, that is, the PCCMM information does not correspond to an underlying TCM formula. Building meaningful TCM formulas from these types of PCCMM information remains an open problem.

## 5 Conclusion

In this study, we constructed the forward and backward feature extraction processes from ACFPs to PCCMM, aiming to identify the implicit medication patterns of the ACFPs published by the Chinese government. We constructed the network from CMM to PCCMM based on ChP as the forward feature extraction process. As the backward process, we introduced constrained combinatorial optimization problems to rebuild the ACFPs from their PCCMMs. The two batches of ACFPs could essentially be rebuilt based on the PCCMM; however, the hyperparameter has a significant impact on the results. We also tested the capability of PCCMM in distinguishing ACFPs from random pseudo-formulas and classifying ACFPs of different deficiency syndromes. In both cases, PCCMM outperformed the nomenclature of CMM as the measurement. The numerical results demonstrated the well-posedness of the CMM–PCCMM network. The PCCMMs facilitate the analysis of TCM formulas, especially ACFPs, from the perspectives of systems science and compressive sensing. High-frequency CMMs were mainly tonic medicines, whereas PCCMMs predominantly mapped to the even–sweet–spleen meridian. The number of associate rules derived from PCCMM significantly surpassed that of those derived from CMM, demonstrating PCCMM’s superiority in uncovering hidden compatibility patterns. Notably, data mining revealed synergistic CMM pairs with low distances and correlations, such as *Jujubae Fructus* (Dazao) and *Zingiberis Rhizoma Recens* (Shengjiang), which aligns with clinical experience and provides data support for the synergistic use of CMMs. Furthermore, we identified negatively correlated CMM pairs and PCCMM pairs, which may guide the discovery of new potential contraindications in CMM combinations. Our study offers a novel method and insights into the mining of ACPFs’ medication patterns and provides clinicians with guidance on TCM formula use and design based on PCCMM.

## Data Availability

The original contributions presented in the study are included in the article/Supplementary Material; further inquiries can be directed to the corresponding author.

## Abbreviations

ACFP, ancient classic and famous prescription; ChP, Chinese Pharmacopoeia; CMM, Chinese medicinal material; PCMM, property of CMM; PCCMM, property combination of CMM; TCM, traditional Chinese medicine; t-SNE, t-distributed stochastic neighbor embedding.
