# Supplementary material for: Understanding the ancient classic and famous prescriptions via the property of Chinese materia medica
Source: Front Pharmacol. 2025 May 12;16:1551531. doi: 10.3389/fphar.2025.1551531 (PMC12104179; doi:10.3389/fphar.2025.1551531)
Supplement: Supplementary file 1 [file Supplementaryfile1.docx]

**Supplementary Figure S1**


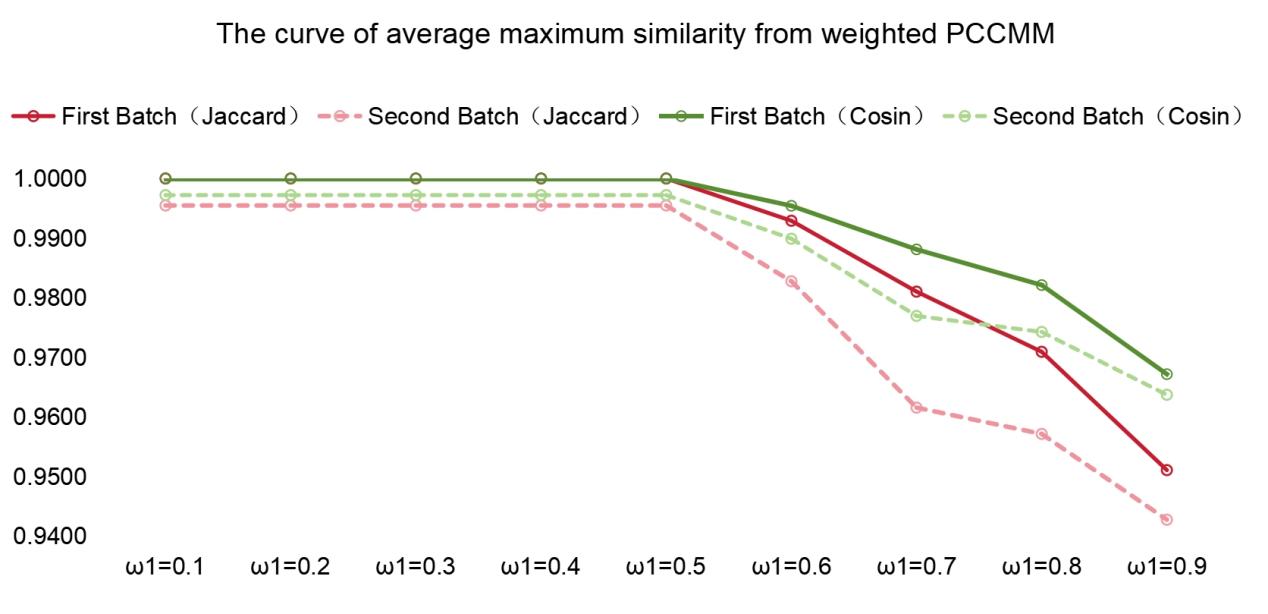


**Supplementary Figure S1.** The Jaccard (red curves) and cosine (green curves) similarties between the ACFPs and the reconstructed TCM formulas based on weighted PCCMM. The solid and dash curves are corresponding to the first and second batches of ACFPs datasets, respectively.

**Supplementary Figure S2**


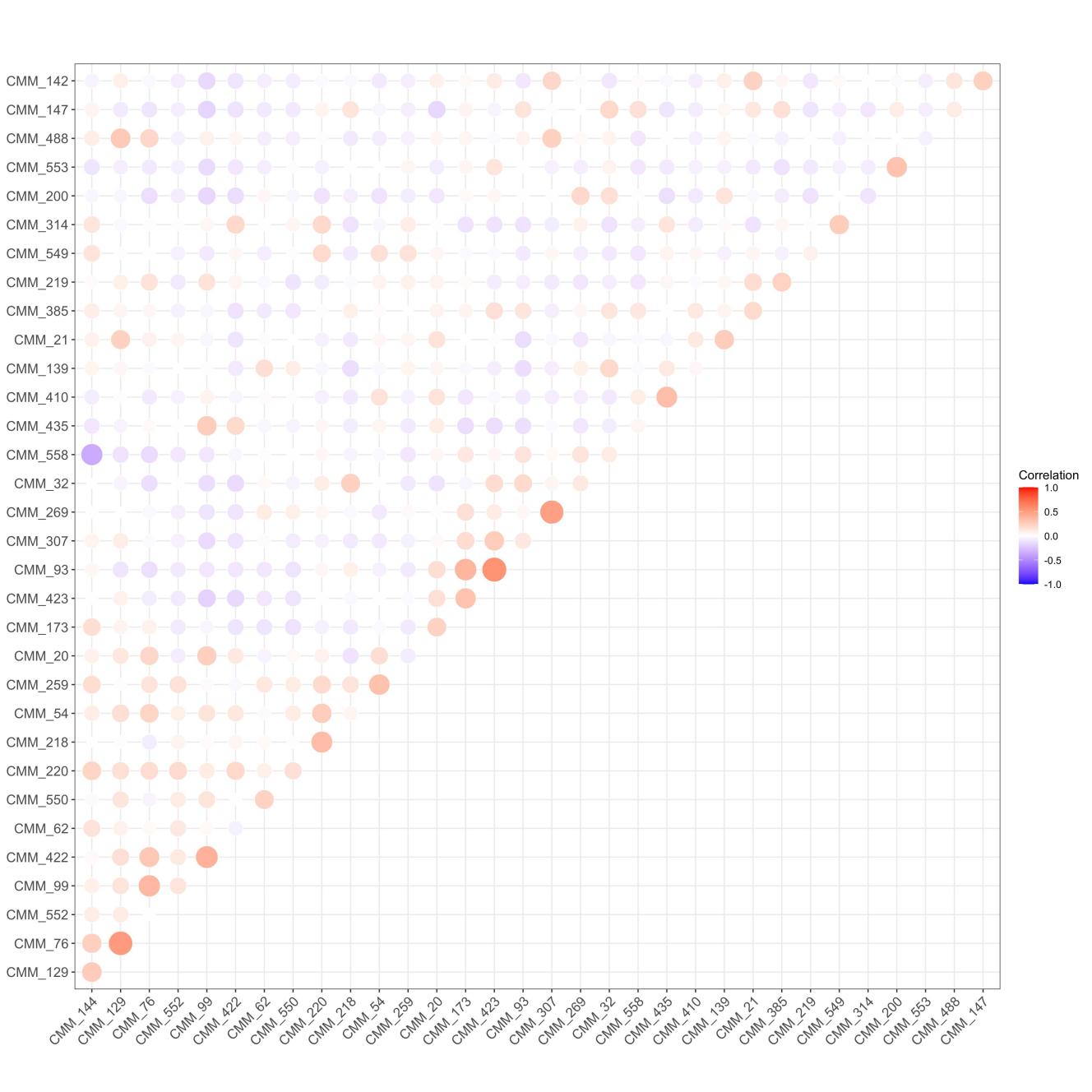


**Supplementary Figure S2.** The correlation of CMM-pairs of ACFPs based on the Pearson correlation coefficient. The larger the circles, the stronger the correlation between the pairs, with blue indicating a negative correlation and red indicating a positive correlation.

**Supplementary Figure S3**


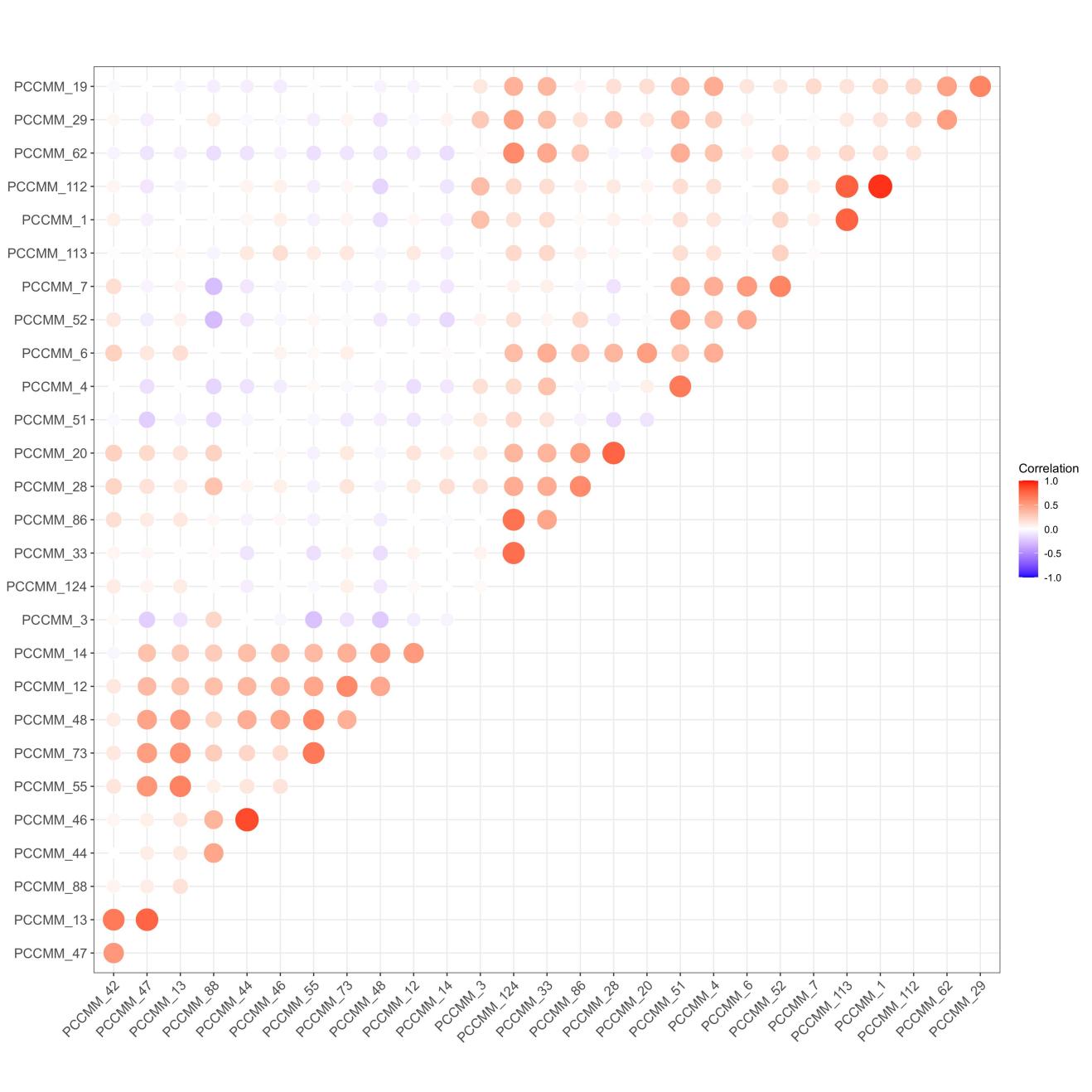
**Supplementary Figure S3.** The correlation of PCCMM-pairs of ACFPs based on the Pearson correlation coefficient. The larger the circles, the stronger the correlation between the pairs, with blue indicating a negative correlation and red indicating a positive correlation.
